# Profiles of Approaches to Writing and Their Links to Self-Efficacy and LLM Acceptance in L2 Academic Writing

**DOI:** 10.3390/bs15070983

**Published:** 2025-07-20

**Authors:** Fei Sun, Laura Mendoza, Junju Wang, Hongbin Li

**Affiliations:** 1 School of Foreign Languages and Literature, Shandong University, Jinan 250100, China; feisun@mail.sdu.edu.cn; 2 Language Centre, University of Helsinki, P.O. Box 4, 00014 Helsinki, Finland; laura.mendoza@helsinki.fi; 3 School of Foreign Languages, Tianjin University, Tianjin 300354, China; xlihb@tju.edu.cn

**Keywords:** approaches to writing, writing self-efficacy, large language model acceptance, L2 academic writing

## Abstract

Approaches to writing play an important role in both the writing processes and outcomes. However, little is known about whether L2 writers adopt different combinations of approaches in academic writing contexts and what factors predict such combinations. Hence, this study aimed to identify different profiles of approaches to writing in an L2 academic context and examine how they are predicted by writing self-efficacy and large language model (LLM) acceptance. To this end, a total of 578 Chinese graduate students were recruited to participate in the study. Latent profile analysis revealed three distinct writing profiles: unorganized (Profile 1), dissonant (Profile 2), and deep and organized (Profile 3), with the majority of students categorized under the dissonant profile. Additionally, multinomial logistic regression analysis revealed that writing self-efficacy positively predicted profile membership, with the strongest effect observed for Profile 3, followed by Profile 2 and then Profile 1. LLM acceptance also positively predicted profile membership, with the strongest effect for Profile 2, followed by Profile 3 and then Profile 1.

## 1. Introduction

Commonly categorized into three types, deep, unreflective, and organized, approaches to writing are closely related to writing performance (Dahl et al., 2023). In academic writing, the deep approach is considered essential, as writers need to synthesize and integrate information from multiple sources to construct coherent and meaningful arguments (Guo et al., 2022). Previous studies show that the deep approach to writing is positively associated with better writing performance (Mendoza et al., 2022), greater well-being (Lonka et al., 2019), and reduced burnout (Yin et al., 2024). Furthermore, the organized approach is positively associated with writing performance, whereas the unreflective approach is negatively associated (Mendoza et al., 2022).

Previous research has investigated students’ approaches to writing in different educational contexts (e.g., Dahl et al., 2023; Lavelle et al., 2013) and with respect to various influencing factors, such as teachers’ assessment methods (Gebrekidan & Zeru, 2023), supervisory feedback (Lonka et al., 2019), and writing self-efficacy (Mendoza et al., 2022). However, most existing studies assume that learners rely on only one dominant approach at a time, such as deep or unreflective, while overlooking the possibility that students may adopt multiple approaches simultaneously in varying combinations. This limitation is particularly salient in the context of L2 academic writing, which involves distinct cognitive, linguistic, and rhetorical demands (Teng & Wang, 2023) and may give rise to more complex combinations of approaches to writing. Moreover, factors such as writing self-efficacy (Mendoza et al., 2023) and emerging technologies like large language models (LLMs) (Kim et al., 2025) are increasingly recognized as influential in this context. However, little is known about how these factors relate to how students combine different approaches to writing.

Therefore, this study aims to investigate the combinations of approaches to writing that students adopt in the L2 academic writing context and examine their relationships with writing self-efficacy and LLM acceptance. It is hoped that this will expand the theoretical understanding of approaches to writing and offer practical implications for designing targeted pedagogical strategies to support students’ L2 academic writing development.

## 2. Literature Review

### 2.1. Approaches to Writing

Approaches to writing refer to how students regulate their cognitive and strategic engagement with writing tasks (Biggs, 1988). Grounded in the broader framework of approaches to learning, they are typically categorized into three types: deep, unreflective, and organized. The deep approach to writing is characterized by active engagement, knowledge transformation, and metacognitive strategy use, while the unreflective approach to writing is marked by passive involvement, knowledge listing, and reproductive thinking (Binks et al., 2022; Lavelle & Bushrow, 2007). Unlike these two approaches, which focus on how students intend to engage with the content of writing tasks, the organized approach emphasizes self-regulation, particularly in terms of time and effort management (Biggs, 1988).

Approaches to writing vary across individuals and contexts (Biggs, 1988). An increasing number of studies have explored contextual and individual factors associated with approaches to writing, including dyslexia and authorial identity (Kinder & Elander, 2012), academic environment (Lonka et al., 2019), supervisory and peer support (Mendoza et al., 2022), assessment methods (Gebrekidan & Zeru, 2023), perceived task difficulty (Chou, 2023), and self-efficacy beliefs (Yin et al., 2024). It has also been found that approaches to writing differ across educational levels (e.g., Lavelle et al., 2002, 2013; Dahl et al., 2023). In terms of writing proficiency, novice writers are more likely to adopt the unreflective approach to writing, while more experienced writers tend to employ the deep approach to writing (Scardamalia & Bereiter, 1987; Teng & Yue, 2023). Moving beyond variable-centered approaches that focus on general associations, recent studies have employed person-centered methods to examine how students combine multiple approaches to writing. For example, Mendoza et al. (2022) identified three distinct profiles of thesis writing approaches among L1 and L2 master’s students in Finland: dissonant, deep and organized, and unorganized. The dissonant profile reflects a conflicting pattern in which students simultaneously demonstrate high levels of both deep and unreflective approaches.

### 2.2. Writing Self-Efficacy and Approaches to Writing

Self-efficacy, rooted in Bandura’s (1986) social cognitive theory, refers to individuals’ belief in their ability to succeed or attain desired outcomes (Bandura, 1997). In L2 learning contexts, it has been shown to be a pivotal factor, predicting learner engagement (Derakhshan & Fathi, 2024), well-being (Huang et al., 2024), academic achievement (Fathi et al., 2024), and informal digital language learning beyond English (G. L. Liu et al., 2024c). Given its skill-specific nature (Bong & Clark, 1999), recent research has increasingly focused on self-efficacy related to particular L2 skills, such as writing. In this context, writing self-efficacy has also been identified as a strong predictor of writing performance (Teng & Yang, 2023; Mendoza et al., 2023).

Prior studies have demonstrated a close relationship between writing self-efficacy and students’ approaches to writing. For instance, Mendoza et al. (2022) found that among Finnish EFL master’s students, writing self-efficacy was positively associated with both deep and organized approaches and negatively associated with the unreflective approach. Zhou et al. (2022), in a study of Chinese EFL high school students, reported a positive relationship between writing self-efficacy and writing engagement, a core characteristic of the deep approach to writing (Lavelle & Bushrow, 2007). Similarly, other studies involving Chinese EFL university students, such as J. Chen et al. (2022), Shen et al. (2024), and Teng (2025), have shown that writing self-efficacy was positively related to the use of metacognitive strategies in writing, another key characteristic of the deep approach to writing (Lavelle & Bushrow, 2007).

### 2.3. LLM Acceptance and Approaches to Writing

LLM acceptance in L2 contexts refers to learners’ willingness to adopt and effectively utilize LLMs in their L2 learning practices (Huang et al., 2024). With the expanding integration of LLMs into language education, research has increasingly examined learner acceptance and usage across a range of formal (e.g., D. Chen et al., 2024; Huang et al., 2024) and informal language learning contexts (e.g., G. Liu & Ma, 2024; G. L. Liu et al., 2024a, 2024b, 2025). More recently, scholarly interest has extended to skill-specific domains such as L2 writing (e.g., Kim et al., 2025; Zou & Huang, 2023). Research in this area has primarily investigated the antecedents of LLM acceptance, including perceived usefulness, ease of use, and learner attitudes (Salam, 2025; Zou & Huang, 2023). In parallel, studies have also highlighted a range of positive outcomes associated with LLM use in writing, such as enhanced writing skills (Fathi & Rahimi, 2024), increased motivation (Song & Song, 2023), and improved performance (A. Nguyen et al., 2024).

Although no direct relationship between LLM acceptance and approaches to writing has yet been established, preliminary evidence shows that LLM acceptance is associated with engagement and the use of metacognitive strategies, suggesting a potential link between the two. For example, Rad et al. (2024) found that Persian EFL students who accepted and effectively leveraged LLMs for English writing tended to exhibit higher levels of writing engagement. A similar pattern was observed among Vietnamese EFL university students (L. Q. Nguyen et al., 2024). In addition, Teng (2025) reported that Chinese EFL university students who accepted and utilized ChatGPT for writing feedback tended to enhance their use of metacognitive strategies. Supporting this, Kim et al. (2025), in a qualitative study, also found that LLMs contributed to the development of metacognitive strategies among Chinese EFL university students.

### 2.4. The Present Study

Prior research on approaches to writing has revealed two primary gaps. First, most existing studies have relied on variable-centered approaches, such as regression and path analysis, which may overlook individual differences in response patterns (Wang et al., 2024). Given that students’ approaches to writing vary across individuals, a person-centered approach such as latent profile analysis (LPA) may offer more nuanced insights by identifying subgroups (profiles) with similar patterns across multiple dimensions (Francot et al., 2021). Students’ classification into these profiles, known as profile membership, can then serve as an outcome variable to examine how individual or contextual factors predict it. Second, research on approaches to writing and their influencing factors has been conducted primarily among students in general writing contexts, with scant attention paid to graduate students engaged in L2 academic writing. Given the unique cognitive and linguistic challenges of this context (Teng & Wang, 2023), it is both timely and necessary to investigate students’ approaches to writing and their predictors, particularly writing self-efficacy and LLM acceptance, within this population.

Based on the above considerations, this study aims to explore profiles of graduate students’ approaches to writing and investigate the roles of writing self-efficacy and LLM acceptance in predicting profile membership in the L2 academic writing context. Specifically, it tries to address the following research questions:

RQ1: What are the profiles of Chinese graduate students’ approaches to L2 academic writing?

RQ2: To what extent do writing self-efficacy and LLM acceptance predict profile membership?

## 3. Method

### 3.1. Context and Participants

The present study was situated within the context of four universities in China. One university was a top-tier institution included in the national “985 Project,” two were national key universities affiliated with the “211 Project,” and one was an institution outside both projects. These universities also differed in academic orientation: one focused on the humanities and social sciences, one specialized in science and engineering, and the remaining two were comprehensive universities covering a wide range of disciplines. Despite these differences, all four universities, like other universities across the country, offered compulsory English courses for graduate students, which put much emphasis on training in academic English writing.

Selected from a convenience sampling method, a total of 578 Chinese graduate students from these four Chinese universities participated in this study. To be eligible, participants had to meet threefold criteria: (1) they were currently enrolled graduate students in Chinese universities; (2) they were learning English as a foreign language; and (3) they had prior experience using LLMs, such as ChatGPT or similar AI tools, for English academic writing tasks. Among them, 323 (55.88%) were female, and 255 (44.12%) were male; 342 (59.2%) were master’s students, and 236 (40.8%) were doctoral students. The age distribution was as follows: 17 students (2.94%) were under the age of 22, 393 (68%) were between 22 and 27, and 168 (29.06%) were over the age of 28. Participants represented diverse academic disciplines, with 330 (57.09%) from the natural sciences and engineering, 193 (33.39%) from the social sciences and humanities, and 55 (9.52%) from other fields. In terms of language background, all participants identified English as their foreign language and had been learning it for at least fourteen years. They all had passed the College English Test Band 6 (CET-6). Additionally, they had received instruction in academic English writing and reported regularly using LLMs to support their English academic writing tasks.

### 3.2. Measures

A composite questionnaire was adopted in this study. The first part of the questionnaire pertains to the demographic information of the participants. The second part consists of three structured scales designed to measure students’ approaches to writing, writing self-efficacy, and LLM acceptance. All items on the scales were rated on a 5-point Likert scale, ranging from 1 (strongly disagree) to 5 (strongly agree).

The scale of approaches to writing included 11 items adapted from Mendoza et al. (2022) to measure students’ approaches to L2 academic writing. It was designed to cover three dimensions, namely, the deep approach to writing (5 items), the unreflective approach to writing (3 items), and the organized approach to writing (3 items). One example item is “While writing my journal article in English, I often contemplate the ideas from multiple perspectives”.

The writing self-efficacy scale had five items adapted from Mendoza et al. (2022) to assess students’ self-efficacy in L2 academic writing. One sample item is “I believe I will finish my journal article in English as planned”.

A 15-item technology acceptance model (TAM) scale, adapted from G. L. Liu et al. (2024a), was used to measure students’ acceptance of LLMs in the L2 academic writing context. The scale included four dimensions: perceived ease of use (3 items), perceived usefulness (5 items), intention to use (3 items), and actual use (4 items). One example item is “I think LLMs can help me write journal articles in English more effectively”.

### 3.3. Data Collection

Data for the study were collected from October to November 2024. Firstly, the researchers contacted English teachers at several universities for their support in distributing a link to an online questionnaire, available on Wenjuanxing (www.wjx.cn, accessed on 23 October 2024), among their students. On the first page of the questionnaire, participants were informed of the study’s purpose, confidentiality measures, and their right to withdraw at any time without penalty. Informed consent was obtained through their action of proceeding to the next page. In total, 643 questionnaires were collected; of these, 65 (10.1%) were excluded due to inconsistent responses or incomplete answers. The remaining 578 (89.9%) valid responses were retained for further analysis.

### 3.4. Data Analysis

Data for the study were analyzed in five steps. First, confirmatory factor analysis (CFA) was performed using AMOS 26.0 to test the structure of the measurement model. Second, composite reliability (CR), average variance extracted (AVE), and the square roots of AVE were calculated using AMOS 26.0 to assess composite reliability, convergent validity, and discriminant validity. Cronbach’s α coefficients were used to evaluate internal consistency. Third, descriptive statistics and Pearson correlation coefficients were obtained using SPSS 27.0. Fourth, LPA was conducted using Mplus 8.0. Model fit was evaluated based on several indices, such as Akaike Information Criteria (AIC), Bayesian Information Criteria (BIC), the adjusted Bayesian Information Criterion (aBIC), the *p*-values of the Lo–Mendell–Rubin Likelihood Ratio Test (LMR) and Bootstrap Likelihood Ratio Test (BLRT), entropy, and class size per profile (Gabriel et al., 2015; Nylund et al., 2007). Models with one to five profiles were compared to determine the best fit. Finally, multinomial logistic regression was performed using SPSS 27.0 to examine the predictive effects of writing self-efficacy and LLM acceptance on students’ profile membership.

## 4. Results

### 4.1. Results of Validity and Reliability Analyses

The initial validity test revealed that the standardized factor loading of the first item under the deep approach to writing dimension (“I put a lot of effort into my journal article in English”) was below the acceptable threshold of 0.50 (Hair et al., 2021). This item was, therefore, removed. After the removal, the remaining items had standardized factor loadings from 0.630 to 0.925. The AVE values for all variables exceeded 0.50, and CR values were above 0.70 (Hair et al., 2021), indicating good convergent validity and composite reliability (see Table 1). The square root of each variable’s AVE was higher than its correlations with other variables, supporting discriminant validity (Fornell & Larcker, 1981). Cronbach’s α values ranged from 0.793 to 0.960, exceeding the recommended threshold of 0.70 (Kline, 2016), showing good internal consistency. The measurement model exhibited acceptable fit indices (χ^2^/*df* = 3.971, RMSEA = 0.072, CFI = 0.921, TLI = 0.909, IFI = 0.921), as recommended by Kline (2016).

### 4.2. Results of Descriptive Statistics and Correlation Analysis

Table 2 presents the results of descriptive statistics and Pearson correlation analyses. Among all examined variables, the level of the deep approach to writing was the highest (M = 3.795, SD = 0.637), and the level of the unreflective approach was the lowest (M = 3.020, SD = 0.932). The SD of these variables ranged from 0.637 to 0.932, indicating moderate variability. Skewness and kurtosis values met normality criteria, with skewness and kurtosis values within the acceptable range of ±3 and ±10, respectively (Kline, 2016). All variables were significantly correlated with one another, except for LLM acceptance and the unreflective approach to writing.

### 4.3. Results of Latent Profile Analysis

**Figure 1:**
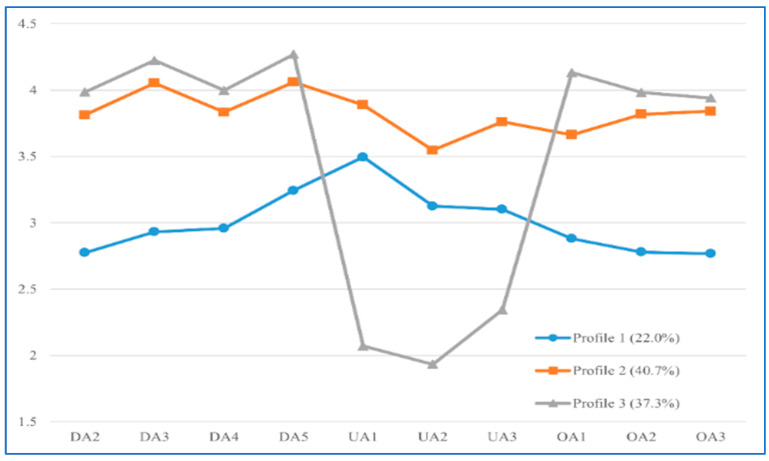
Mean scores of approaches to writing dimensions by profile. Note: DA = deep approach to writing; UA = unreflective approach to writing; OA = organized approach to writing.

Table 3 presents the fit indices for each model with a different number of profiles, ranging from one to five. The best-fitting model was selected based on higher entropy, lower AIC, BIC, and aBIC values, significant *p*-values (*p* < 0.05) for the LMR and BLRT tests (Gabriel et al., 2015), and adequate class sizes (i.e., each profile representing more than 5% of the total sample) (Nylund et al., 2007). Based on these criteria, the three-profile model was chosen as the optimal solution. As shown in Table 4, the diagonal values of the posterior probability matrix indicated that the average probability of membership in each profile exceeded 90%, suggesting that most participants were accurately classified. The characteristics of the three profiles are illustrated in Figure 1. Following their characteristics and drawing on prior literature (Mendoza et al., 2022), the profiles were named as follows: unorganized profile (Profile 1, N = 127, 22.0%), dissonant profile (Profile 2, N = 240, 40.7%), and deep and organized profile (Profile 3, N = 211, 37.3%), respectively.

### 4.4. Results of Multinomial Logistic Regression Analysis

Table 5 presents the results of the multinomial logistic regression analysis, which examines the predictive effects of writing self-efficacy and LLM acceptance on profile membership. The unorganized profile was used as the reference group. The odds ratio (OR) indicates how a one-unit increase in a predictor variable affects the likelihood of membership in a specific profile, compared to the reference profile. An OR greater than 1 indicates increased odds of membership in that profile relative to the reference group, while an OR less than 1 indicates decreased odds. Specifically, a one-unit increase in writing self-efficacy was significantly associated with higher odds of being classified into the dissonant profile (OR = 7.942, *p* < 0.001) and the deep and organized profile (OR = 27.020, *p* < 0.001), compared with the unorganized profile. This suggests that, among the three profiles, writing self-efficacy had the strongest predictive effect for membership in the deep and organized profile, followed by the dissonant profile, and weakest for the unorganized profile. Similarly, a one-unit increase in LLM acceptance also significantly increased the odds of membership in both the dissonant profile (OR = 2.526, *p* < 0.001) and the deep and organized profile (OR = 1.675, *p* < 0.05), relative to the unorganized profile. These results indicate that LLM acceptance had the strongest predictive effect for the dissonant profile, followed by the deep and organized profile, and weakest for the unorganized profile.

## 5. Discussion

### 5.1. Profiles of Approaches to Writing

This study identified three distinct profiles of writing approaches among Chinese graduate students engaged in L2 academic writing: unorganized, dissonant, and deep and organized. The results suggest that these students tend to adopt multiple writing approaches simultaneously and that these approaches are combined in different patterns. The profiles identified in this study align with those found in research on graduate students’ approaches to thesis writing in Finland (Mendoza et al., 2022). While Mendoza et al.’s (2022) study encompassed both L1 and L2 academic writing, the present study focuses specifically on L2 academic writing, thereby extending previous findings to a more targeted context.

Within the first profile, the unorganized profile (N = 211, 37.3%), students exhibited the lowest levels of both deep and organized approaches to writing, along with a moderate level of unreflective approach to writing. This suggests that they lack the ability to construct coherent texts and to manage their writing process effectively. The identification of this profile aligns with findings in the writing domain (Mendoza et al., 2022) but contrasts with learning-focused studies, where such a profile has not been observed (e.g., Asikainen et al., 2020; Parpala et al., 2022; Tuononen et al., 2023). This difference suggests that, although approaches to writing are conceptually grounded in the broader framework of approaches to learning (Biggs, 1988), the way students combine these approaches may vary across contexts. One possible explanation lies in the nature of writing itself. Writing is a cognitively demanding task (Reynolds & Teng, 2021) that requires coordination of planning, translating, and reviewing processes (Hayes, 2012). These demands may make organizational difficulties more visible in writing, contributing to the emergence of this profile.

A notable and unexpected finding is that the dissonant profile accounted for the largest proportion of the sample (41.5%, N = 240). Students in this group exhibited the second highest levels of deep and organized approaches to writing, along with the highest level of unreflective approach. This suggests that, while they aim to write in a structured and meaningful way, they still struggle with fragmented writing. This contrasts with Mendoza et al. (2022), where the dissonant profile represented the smallest proportion (20.5%) of Finnish thesis writers. One explanation may lie in language background. Mendoza et al.’s (2022) study included both L1 and L2 thesis writers, while the present study focused exclusively on L2 academic writing, which is more demanding due to linguistic barriers and higher cognitive load (Kim et al., 2025; Scardamalia & Bereiter, 1987; Teng & Wang, 2023). The Chinese educational context may also play a role. With its exam-oriented, grammar-focused instruction (Yan, 2012), students may overemphasize linguistic form at the expense of content and ideas (X. Zhang & Hadjioannou, 2022), resulting in mixed and conflicting writing approaches. As a result, Chinese students may exhibit conflicting tendencies: aspiring to write in a deep and organized manner while still relying on the unreflective approach.

In line with expectations, the third profile, deep and organized (N = 211, 37.3%), was marked by the highest levels of deep and organized approaches to writing and the lowest unreflective approach. This suggests that these students aim to produce coherent texts while effectively managing their time and structuring their writing process. In contrast to the dissonant profile, which combines the second-highest levels of deep and organized approaches with the highest level of unreflective writing, the deep and organized profile represents a more coherent integration of different approaches to writing. This finding aligns with prior variable-centered research showing that deep and organized approaches are positively associated in both general learning (Herrmann et al., 2017; Yin et al., 2022) and writing contexts (Mendoza et al., 2022). It also echoes evidence from previous studies indicating that both deep and organized approaches are negatively related to the unreflective approach in learning contexts (Tuononen et al., 2023). By adopting a person-oriented approach, the present study extends prior findings to the L2 academic writing context among Chinese graduate students.

### 5.2. The Predictive Roles of Writing Self-Efficacy and LLM Acceptance

The results of the study indicated that both writing self-efficacy and LLM acceptance were significant predictors of profile membership, although their predictive strengths varied. These findings are consistent with the framework of approaches to writing, which posits that approaches to writing emerge from the interaction between the writer and the writing environment (Biggs, 1988; Lavelle & Bushrow, 2007).

Writing self-efficacy was significantly and positively associated with profile membership. Specifically, writing self-efficacy was the strongest predictor of membership in the deep and organized profile, followed by the dissonant profile, and was weakest for the unorganized profile. This implies that Chinese graduate students with the highest levels of writing self-efficacy in L2 academic writing are more likely to be classified into the deep and organized profile, those with moderate levels into the dissonant profile, and those with the lowest levels into the unorganized profile. This finding is consistent with previous research, which has shown that writing self-efficacy is positively associated with deep and organized approaches to writing and negatively associated with the unreflective approach to writing (Mendoza et al., 2022). Similar associations have also been observed in broader L2 learning contexts, where self-efficacy has been found to positively predict the deep approach to learning (Sun & Shi, 2024; Zhan et al., 2021).

Differences in metacognitive strategy use may help explain why writing self-efficacy predicts profile membership. Learners with the highest levels of writing self-efficacy tend to actively employ metacognitive strategies, such as planning, monitoring, reflecting, and evaluating (Shen et al., 2024; Teng, 2025). These metacognitive strategies enable them to structure their ideas coherently, reflect critically on their arguments, and make purposeful revisions, thereby increasing their likelihood of being classified into the deep and organized profile. Conversely, learners with the lowest levels of writing self-efficacy have been found to use metacognitive strategies less frequently (Shen et al., 2024; Teng, 2025), which may result in limited planning and minimal reflective processing, features commonly associated with the unorganized profile. Those with moderate self-efficacy also tend to demonstrate a moderate level of metacognitive strategy use (J. Chen et al., 2022). However, this may not be sufficient to sustain consistent application of these metacognitive strategies throughout the writing process, which could explain their likelihood of falling into the dissonant profile.

LLM acceptance was also significantly and positively associated with profile membership. Specifically, students with higher levels of LLM acceptance were most likely to belong to the dissonant profile, followed by the deep and organized profile, and least likely to be classified into the unorganized profile. This suggests that Chinese graduate students with the highest levels of LLM acceptance in the L2 academic writing context are more likely to be classified into the dissonant profile, those with moderate levels into the deep and organized profile, and those with the lowest levels into the unorganized profile. This finding partially aligns with previous variable-centered studies, which reported that technology acceptance is positively related to the deep approach to learning and negatively related to the unreflective approach to learning (Chan & Hu, 2023; Okur & Hamutoğlu, 2023). This finding also echoes earlier research demonstrating positive associations between LLM acceptance to metacognitive strategy use (Teng, 2025), one key feature typically associated with the deep approach to writing.

One possible explanation for the varying predictive strength of LLM acceptance lies in the dual effects of LLMs. Students with higher acceptance may benefit from timely feedback and enhanced support (Song & Song, 2023) but may also risk developing an overreliance on these tools, turning to LLMs for quick answers rather than engaging in independent problem-solving (L. Zhang & Xu, 2025). Such overreliance has been shown to hinder cognitive development (Zhai et al., 2024) and writing skills acquisition (L. Zhang & Xu, 2025). In contrast, students with low LLM acceptance may lack adequate scaffolding, increasing the likelihood of falling into the unorganized profile. Those with moderate acceptance may strike a balance, benefiting from LLM support while maintaining cognitive engagement and autonomy, contributing to their classification into the deep and organized profile. However, since LLM overreliance was not directly measured in this study, these interpretations remain conjectural and should be empirically tested in future research.

## 6. Implications and Limitations

This study has both theoretical and pedagogical implications. Theoretically, its contributions lie in three aspects. First, it extends the framework of approaches to writing to the L2 academic writing context of Chinese graduate students, confirming its applicability beyond Western academic contexts. Second, it highlights variation in students’ approaches to L2 academic writing, offering a more nuanced understanding of how multiple approaches coexist across learners. Third, by introducing LLM acceptance as a predictor of profile membership, this study expands the framework of approaches to writing to include technological acceptance as a key contextual factor, underscoring the need to consider AI-related variables in theorizing approaches to writing. Pedagogically, the identification of these distinct profiles suggests the value of differentiated instruction, as educators should tailor support to students’ diverse approaches to writing. In particular, as writing self-efficacy most strongly predicts membership in the deep and organized profile, instructional strategies aimed at enhancing students’ writing self-efficacy are essential. Furthermore, given the differential impact of LLM acceptance across profiles, teachers should provide explicit guidance on its ethical and critical use, encouraging students to engage with LLMs thoughtfully rather than dependently.

While this study provides useful implications, several limitations should be noted. First, relying solely on quantitative self-report data limited insights into why students fell into specific profiles, particularly the dissonant group, as well as the mechanisms through which individual or contextual factors exerted their influence. Future research could adopt a mixed-method design by incorporating qualitative data (e.g., open-ended responses or interviews) to gain a more nuanced understanding. Second, key individual and contextual factors, such as LLM overreliance, English learning backgrounds (e.g., proficiency, duration, and writing experience), disciplinary differences, and degree level, were not examined. These unmeasured factors may have influenced profile formation and should be considered in future research. Third, the sample size was relatively modest. Increasing the number of participants from a broader range of universities could strengthen the statistical power and enhance the generalizability of the findings.

## 7. Conclusions

Employing a person-centered approach, this study investigated Chinese graduate students’ approaches to L2 academic writing and their relationships with writing self-efficacy and LLM acceptance. Three distinct profiles are identified: unorganized, dissonant, and organized, with the dissonant profile comprising the largest proportion of participants. Both writing self-efficacy and LLM acceptance significantly predicted profile membership, though their predictive strengths varied across profiles. Writing self-efficacy was the strongest predictor of membership in the deep and organized profile, followed by the dissonant profile, and weakest for the unorganized profile. LLM acceptance most strongly predicted membership in the dissonant profile, followed by the deep and organized profile, and least strongly in the unorganized profile.

## Figures and Tables

**Table 1:** Results of reliability, convergent validity, and discriminant validity analyses.

				The Square Root of AVE and Squared Correlation Coefficient Matrix				
Variable	*α*	CR (>0.70)	AVE (>0.50)	DA	UA	OA	WSE	LLM
DA	0.828	0.831	0.551	**0.743**				
UA	0.840	0.845	0.648	−0.248 ***	**0.805**			
OA	0.793	0.812	0.594	0.818 ***	−0.229 ***	**0.771**		
WSE	0.895	0.896	0.633	0.768 ***	−0.284 ***	0.695 ***	**0.796**	
LLM	0.960	0.928	0.765	0.525 ***	0.006	0.379 ***	0.405 ***	**0.874**

Note: DA = deep approach to writing; UA = unreflective approach to writing; OA = organized approach to writing; WSE = writing self-efficacy; LLM = LLM acceptance. The square root of AVE is demonstrated along the diagonal line in bold; *** *p* < 0.001.

**Table 2:** Results of descriptive statistics and correlation analysis among variables.

Variable	DA	UA	OA	WSE	LLM
DA	1				
UA	−0.171 **	1			
OA	0.714 **	−0.202 **	1		
WSE	0.670 **	−0.231 **	0.629 **	1	
LLM	0.469 **	0.017	0.356 **	0.393 **	1
M	3.795	3.020	3.653	3.745	3.784
SD	0.637	0.932	0.716	0.670	0.673
Skewness	−0.902	−0.034	−0.514	−0.348	−0.529
Kurtosis	3.118	−0.495	1.103	0.786	1.658

Note: DA = deep approach to writing; UA = unreflective approach to writing; OA = organized approach to writing; WSE = writing self-efficacy; LLM = LLM acceptance; ** *p* < 0.01 (2-tailed).

**Table 3:** Results of model fit for the latent profile analyses.

Model	AIC	BIC	aBIC	Entropy	LMR (*p*)	BLRT (*p*)	Profile Sizes
1-Profile	14,976.285	15,063.477	14,999.985				
2-Profile	14,008.101	14,143.248	14,044.836	0.840	0.032	0.000	163/415
3-Profile	13,438.995	13,622.097	13,488.764	0.851	0.025	0.000	127/240/211
4-Profile	13,045.294	13,276.351	13,108.097	0.863	0.086	0.000	11/181/215/171
5-Profile	12,766.527	13,045.540	12,842.365	0.894	0.007	0.000	11/114/200/230/23

**Table 4:** Results of the average posterior probability for latent profile membership.

	1	2	3
1	0.929	0.058	0.013
2	0.031	0.915	0.053
3	0.008	0.038	0.955

**Table 5:** Results of multinomial logistic regression analysis for the effects of predictors on profile membership.

	Dissonant Profile (Profile 2)		Deep and Organized Profile (Profile 3)	
	B (SE)	OR	B (SE)	OR
Writing self-efficacy	2.072 (0.269)	7.942 ***	3.297 (0.304)	27.020 ***
LLM acceptance	0.927 (0.227)	2.526 ***	0.516 (0.236)	1.675 *

Note: The reference group is the unorganized profile (Profile 1); *** *p* < 0.001, * *p* < 0.05.

## Data Availability

The data presented in this study are available upon request from the corresponding author.
